# Associations between Unhealthy Diet and Lifestyle Behaviours and Increased Cardiovascular Disease Risk in Young Overweight and Obese Women

**DOI:** 10.3390/healthcare4030057

**Published:** 2016-08-19

**Authors:** Megan C. Whatnall, Clare E. Collins, Robin Callister, Melinda J. Hutchesson

**Affiliations:** 1 School of Health Sciences, Faculty of Health and Medicine, and Priority Research Centre in Physical Activity and Nutrition, University of Newcastle, Callaghan 2308, Australia; megan.whatnall@uon.edu.au (M.C.W.); clare.collins@newcastle.edu.au (C.E.C.); 2 School of Biomedical Sciences and Pharmacy, Faculty of Health and Medicine, and Priority Research Centre in Physical Activity and Nutrition, University of Newcastle, Callaghan 2308, Australia; robin.callister@newcastle.edu.au

**Keywords:** cardiovascular disease risk, nutrition, lifestyle behaviours, young women

## Abstract

Unhealthy lifestyle behaviours are known modifiable risk factors for cardiovascular disease (CVD). This cross-sectional analysis aimed to describe lifestyle behaviours and CVD risk markers in young overweight and obese Australian women and explore associations between individual and combined lifestyle behaviours with CVD risk markers. Lifestyle behaviours assessed were diet quality, alcohol intake, physical activity, sitting time and smoking status, and were combined to generate a Healthy Lifestyle Score (HLS) (0–5). Objectively measured CVD risk markers were body mass index (BMI), %body fat, waist circumference, blood pressure, and plasma cholesterol and triglycerides. Analysis included 49 women aged 18–35 years, with BMI 25.0 to 34.9 kg/m^2^. The mean ± SD Australian Recommended Food Score was 33.5 ± 9.3 points, alcohol 3.3 ± 2.4 standard drinks/day, physical activity 207 ± 225 min/week and sitting time 578 ± 213 min/day. All participants were non-smokers. The proportion of participants outside normal reference ranges was 83.7% for waist circumference (*n* = 41), blood pressure 0% (*n* = 0), total cholesterol 26.2% (*n* = 11), HDL cholesterol 38.6% (*n* = 17), LDL cholesterol 22.7% (*n* = 10), and triglycerides 4.2% (*n* = 2). Physical activity was inversely associated with body fat (β = −0.011%, *p* = 0.005), diastolic blood pressure (β = −0.010 mmHg, *p* = 0.031) and waist circumference (β = −0.013 cm, *p* = 0.029). Most participants (59.2%, *n* = 29) had a HLS ≤ 2. No significant associations were found between HLS and CVD risk markers. Insufficient physical activity was the primary lifestyle factor associated with increased CVD risk markers, which suggests interventions targeting physical activity in young women may potentially improve cardiovascular health.

## 1. Introduction

Cardiovascular disease (CVD) is the leading cause of total and premature death in women globally [1,2]. Lifestyle behaviours known to increase CVD risk include high alcohol consumption, poor diet quality, physical inactivity, extended sitting time and smoking [3,4,5,6]. The prevalence of these lifestyle behaviours in women is high, with >90% of women in Australia and the USA reporting one or more risk behaviour [6,7]. In 2008, 34% of adult women worldwide were not sufficiently physically active [8], while 53% of 18–34-year-old Australian women are not meeting physical activity recommendations [9]. Most consume inadequate servings of fruits and vegetables, including 96% of 18–34-year-old Australian females [10]. A significant proportion of adult females smoke, at 7% globally [11], 15% in America [12] and 16% in Australia [13]. In the USA, of the 60% of adult women who drink alcohol, 13% consume more than the recommended daily amount [14]. In Australia 10% of adult females exceed national alcohol guidelines of ≤2 drinks/day [15]. Studies of sitting time in US and Australian adult women indicate 51% and 75% respectively of participants spend >3 h sitting/day [5,16].

Several observational studies have demonstrated that higher CVD risk is conferred by a greater number of risky lifestyle behaviours [17,18,19,20,21,22]. For example, a prospective cohort study of Swedish women aged 49–83 years investigated the association between a low risk lifestyle pattern (healthy diet, moderate alcohol consumption, never smoking, being physically active, healthy body mass index (BMI)) and stroke risk. Over the ten-year follow-up, those with all five low risk lifestyle behaviours had a 54% lower risk of stroke than those with none [17]. This relationship is supported by similar studies in middle and older-aged males and females [18,19,20]. Yang et al. examined the association between seven cardiovascular health metrics (not smoking, physically active, normal blood pressure, blood glucose and total cholesterol levels, normal weight and a healthy diet) and CVD mortality in a US adult cohort [20]. Over a median follow-up of 14.5 years, adults with six or more healthy behaviours had an absolute CVD mortality risk of 15% compared to 65% for those reporting only one or no healthy behaviours [20].

O’Flaherty et al. in a time trend analysis of Australian men and women found that the current 30-year trend of declining coronary heart disease (CHD) mortality is slowing, suggesting that young adults will experience greater CHD morbidity and mortality compared to the current generation of older adults [23]. The high prevalence of risk behaviours in young adults currently supports this trend [6,7,8,10,11,12,13,14,15,16,24]. However, few studies have specifically investigated young adults’ lifestyle behaviours and CVD risk. The Coronary Artery Risk Development in Young Adults (CARDIA) study examined whether a healthy lifestyle (never smoking, healthy BMI, no/moderate alcohol intake and higher healthy diet and physical activity scores) in young adulthood (18–30 years) was associated with a low CVD risk profile (no pre-existing CVD, untreated cholesterol <200 mg/dL, untreated blood pressure <120/<80 mmHg, never smoking, and no history of diabetes or myocardial infarction) in middle age [21]. The prevalence of a low CVD risk profile was 60% for those with five healthy lifestyle factors compared to 3% for those with one [21]. Chomistek et al. conducted a similar study in young and middle-aged women (27–44 years at baseline) exploring the association between a healthy lifestyle (no smoking, healthy BMI, higher diet quality, no/moderate alcohol intake, no/moderate television viewing and being physically active) and clinical CVD risk factors (diabetes, hypertension and hypercholesterolaemia) over a 20-year follow-up [22]. They found that 46% of clinical CVD risk factor cases were attributable to a poor lifestyle [22].

One study has specifically explored the direct association between lifestyle behaviours during young adulthood and CVD risk [25]. Gall et al. examined cross-sectionally the association between a healthy lifestyle (healthy BMI, non-smoker, low alcohol, physically active and healthy dietary behaviours) and CVD risk factors (blood pressure, LDL and HDL cholesterol, triglycerides, glucose, insulin and insulin resistance) in young Australian adults (25–36 years) [25]. Significant associations were found between a healthy lifestyle and HDL cholesterol (β = 0.02) and glucose (β = −0.02) in females, with a number of significant associations also found for the male study participants [25]. These findings suggest that unhealthy lifestyle behaviours in young adulthood are having a direct impact on cardiovascular health, and that further exploration of such an association in this age group is needed.

The current study investigated these relationships at baseline in young overweight and obese women (18–35 years) enrolled in a weight-loss study. The first aim was to describe the young women’s lifestyle behaviours (diet quality, alcohol intake, physical activity, sitting time and smoking) and CVD risk markers (BMI, %body fat, waist circumference, blood pressure, total, HDL and LDL cholesterol and triglycerides). The second aim was to examine associations between lifestyle behaviours and CVD risk markers. The final aim was to explore associations between a total Healthy Lifestyle Score (HLS) that summarises overall diet quality, alcohol intake, physical activity, sitting time and smoking status, and CVD risk markers. It was hypothesised that the study population would have unhealthy lifestyle behaviours and elevated CVD risk markers, and that unhealthy lifestyle behaviours and a lower HLS would be associated with higher CVD risk.

## 2. Materials and Methods

### 2.1. Study Design

This study was a cross-sectional analysis of baseline data from the Be Positive Be Healthe randomised controlled trial (RCT), which evaluated efficacy of a weight-loss program targeting young (18–35 years) overweight and obese women.

### 2.2. Participants and Recruitment

RCT inclusion criteria were: female, 18–35 years, BMI 25.0–34.9 kg/m^2^, email and internet access, iPhone model 4s or newer, social media accounts willing to be used for the study and able to attend measurement sessions. Participants were excluded if: currently pregnant or breastfeeding, planning pregnancy in next 6 months, participating in another weight-loss program, taking medications which had caused weight gain, had a metabolic disorder, eating disorder or other medical condition where weight loss may compromise health, non-English speaking or had weight loss of ≥5% of initial weight in the last three months.

Participants were recruited via media releases by the University of Newcastle and Hunter Medical Research Institute, posters at the university campus, local technical college, local businesses and organisations known to engage with the target group, and social media pages of these settings [26]. Email invitation was also sent to consenting participants of a previous research study.

All participants gave written informed consent prior to participating in the study. The study was conducted in accordance with the Declaration of Helsinki and the protocol was approved by the University of Newcastle Human Research Ethics Committee (H-2014-0138).

### 2.3. Measures

An online questionnaire collected self-reported data on dietary and alcohol intakes, physical activity, sitting time and smoking status using Survey Monkey (www.surveymonkey.com.au). Participants attended the University of Newcastle campus for objective measurement of CVD risk markers. The online questionnaires were completed within the two days prior to or during the baseline measurement session.

#### 2.3.1. Exposure: Measurement of Lifestyle Behaviours

Dietary intake was assessed using the validated Australian Eating Survey food frequency questionnaire (AES FFQ) [27], a self-administered 120-item semi-quantitative FFQ which asks respondents to report usual intake over the previous six months. Diet quality was determined using the validated Australian Recommended Food Score (ARFS) [28], calculated using a sub-set of 70 items from the AES FFQ relating to intake of vegetables, fruit, meat/flesh foods, non-meat/flesh protein foods, breads and cereals, dairy foods, water and spreads/sauces. The ARFS adds the points for each item, with most foods attributed one point for a consumption frequency of ≥ once per week, and the total score ranging from zero to 73 points. A higher score is indicative of greater dietary variety, more optimal nutrient intakes [28] and dietary patterns more aligned with the Australian Dietary Guidelines [29].

Alcohol intake was assessed using questions from the New South Wales Adult Population Health Survey [30], based on participants’ alcohol consumption over the previous six months. Participants were asked how many standard drinks they usually consumed on an occasion where they drank alcohol.

Physical activity was assessed using the Godin Leisure Time Exercise Questionnaire [31]. Participants indicated the number of sessions per week and time (minutes) per session spent performing vigorous, moderate and mild intensity physical activity, based on the previous month. Total physical activity (min/week) was calculated as the sum of the number of sessions per week × time per session for vigorous and moderate activity.

Sitting time was assessed using the validated Domain-Specific Sitting Time Questionnaire [32]. Participants were asked to indicate the time (minutes) spent sitting/day in the previous month in five different situations (during travel, at work, watching television, using a computer at home, and in leisure time excluding television viewing), on a weekday and on a weekend day. Total sitting time/day for weekdays and weekend days was determined as the sum of sitting time for each situation. The average sitting time/day was calculated ((total time spent sitting on a weekday × 5) + (total time spent sitting on a weekend day × 2))/7.

Participants were asked if they currently smoke tobacco products, and if so if they have smoked at least 100 cigarettes or a similar amount of tobacco in their life. Participants were classed as a “smoker” if they reported smoking “daily” or smoking “at least once a week” or “less often than once a week” and smoking 100 cigarettes or a similar amount in their life. Participants were otherwise classed as a “non-smoker”.

All five lifestyle behaviours (diet quality, alcohol intake, physical activity, sitting time and smoking) were included in defining the HLS. Similar to a number of previous studies [17,18,19,20,21,22] participants were awarded one point for each healthy lifestyle behaviour, therefore the HLS ranged from zero to five points, with a higher score indicating a healthier lifestyle. Points were attributed based on meeting population-based guidelines if available [33,34]. If no population-based recommendations were available, cut-points were based on the participants’ data, with the highest/lowest quintile used. Therefore, participants were awarded one point for: an ARFS in the top quintile of the distribution of the cohort, alcohol intake of ≤2 standard drinks on a day when alcohol is consumed [33], an accumulated 150 min of moderate, 75 min of vigorous or equivalent combination of moderate and vigorous physical activity/week [35], average sitting time in the lowest quintile of the distribution of the cohort, and if they were a non-smoker.

#### 2.3.2. Outcomes: Cardiovascular Disease (CVD) Risk Markers

Height was measured to 0.1 cm using the stretch stature method on a stadiometer (Inbody BSM370; Inbody Australia, Miami, QLD, Australia). Weight to 0.01 kg was measured in light clothing, without shoes on a digital scale and body fat percentage determined using bioelectrical impedance (Inbody 720; Inbody Australia, Miami, QLD, Australia). BMI was calculated using the standard equation (weight (kg)/height (m^2^)) [36]. Waist circumference was measured to 0.1 cm using a non-extensible steel tape measure as the narrowest point between the lower costal border and the umbilicus. Blood pressure was measured using the automatic sphygmomanometer (Inbody BPBIO320, Inbody Australia, Miami, QLD, Australia). Participants were seated for five minutes prior to the first blood pressure measurement, with two minutes rest between additional measures. Total, HDL and LDL cholesterol and triglycerides were measured in a fasting blood sample (minimum 8 h, maximum 12 h). Blood samples were obtained by finger prick and analysed using the validated Cardiochek reflectance spectrophotometer lipid measurement tool (Point of Care Diagnostics Pty Ltd, Artarmon, NSW, Australia) [37,38]. All measurements for height, weight, waist circumference and blood pressure were taken twice for accuracy, with a third measurement also taken in cases where either of the first two values fell outside a predetermined acceptable range.

#### 2.3.3. Socio-Demographic Characteristics

Socio-demographic data were collected including age, education, qualifications, income, ethnicity and postcode. Postcode of residence was used as an indicator of socio-economic status based on the Australian Bureau of Statistics Index of Relative Socio-Economic Advantage and Disadvantage (IRSAD) [39].

### 2.4. Statistical Analysis

Data analysis was conducted using Stata statistical software version 14.1 (StataCorp, College Station, TX, USA). Descriptive statistics were used to describe lifestyle behaviours, CVD risk markers and socio-demographic variables including standard deviations (SD) and means for continuous variables, and percentages for categorical variables. Unadjusted linear regression models were used to explore the association between individual lifestyle behaviours (diet quality, alcohol intake, physical activity and sitting time) and each CVD risk marker (BMI, %body fat, waist circumference, blood pressure, total, HDL and LDL cholesterol and triglycerides). Each linear regression model was then repeated (adjusted model), to also include the other lifestyle behaviours, due to potential confounding. Finally, linear regression was used to explore the association between HLS and each CVD risk marker. All linear regression models were controlled for age, ethnicity and socio-economic status. Statistical significance was set at *p* < 0.05.

## 3. Results

### 3.1. Socio-Demographic Status

A total of 57 women were recruited, with 49 included in the analyses. The remaining were excluded due to missing physical activity data (*n* = 5) or implausible sitting time data (*n* = 3). Participants’ sociodemographic characteristics are summarised in Table 1. The mean ± SD age of participants was 27.1 ± 4.5 years. The majority was currently studying (55.1%), and were university students (53.1%). The greatest proportion of participants had completed a university or higher university degree (42.9%), followed by a certificate/diploma (26.5%). The majority were born in Australia (93.9%), including 2.0% of Aboriginal descent. Most (42.9%) reported a middle-range income ($300–$999/week), with a similar proportion (44.9%) rating in the middle range (4–6) on the IRSAD.

### 3.2. Lifestyle Behaviours

Table 1 also summarises data for the five lifestyle behaviours. The mean ± SD ARFS was 33.5 ± 9.3 points. The cut-point for the top quintile was ≥40 points, with 75.5% scoring ≤40 points. The mean number of standard drinks consumed/day, on days when alcohol was consumed, was 3.3 ± 2.4 standard drinks, with 44.9% exceeding recommendations of ≤2 standard drinks on any day when alcohol was consumed. The mean total physical activity (minutes/week) was 207 ± 225, with almost half (49.0%) reporting insufficient physical activity. The mean time spent sitting was 499 ± 200 min on a weekend day and 610 ± 260 min on a weekday, with an average sitting time of 578 ± 213 min/day. The cut-point for the lowest quintile of sitting time was ≤394 min/day. Based on the average sitting time, 81.6% of participants sat for ≥394 min/day. All participants were non-smokers.

### 3.3 CVD Risk Markers

Table 1 summarises data on CVD risk markers. The mean BMI was 29.4 ± 2.6 kg/m^2^, with 44.9% of participants obese. Mean %body fat was 37.7% ± 5.7%. Mean waist circumference was 88.8 ± 8.6 cm, with most participants (83.7%) being rated at increased risk of metabolic complications (≥80 cm), and close to half (49.0%) at substantially increased risk of metabolic complications (≥88 cm) [40]. The mean systolic blood pressure was 112 ± 9 mmHg, and 74 ± 8 mmHg for diastolic. No participants had hypertension (>140/90 mmHg), however 10.0% had blood pressure in the normal–high range (>120/80 mmHg) [41]. Mean total cholesterol was 5.0 ± 0.9 mmol/L, with 33.3s% of plasma total cholesterol concentrations ≥5.5 mmol/L [42]. Mean HDL cholesterol was 1.5 ± 0.4 mmol/L, with 38.6% of plasma HDL cholesterol concentrations <1.3 mmol/L [42]. Mean LDL cholesterol was 2.9 ± 0.8 mmol/L, with 22.7% of participants with concentrations ≥3.5 mmol/L [42]. The mean plasma triglyceride concentration was 1.2 ± 0.5 mmol/L, with 4.2% of participants triglycerides ≥2.0 mmol/L [43].

### 3.4. Linear Regression Analysis of Lifestyle Behaviours with CVD Risk Markers

Linear regression of individual lifestyle behaviours and CVD risk markers are presented in Table 2. In the unadjusted models, physical activity was significantly and inversely associated with %body fat (β = −0.011%, *p* = 0.005), waist circumference (β = −0.013 cm, *p* = 0.029) and diastolic blood pressure (β = −0.010 mmHg, *p* = 0.031). A significant inverse association was also identified between alcohol intake and BMI (β = −0.458, *p* = 0.012), waist circumference (β = −1.673 cm, *p* = 0.003) and diastolic blood pressure (β = −0.988 mmHg, *p* = 0.039). In the adjusted models, controlling for the other lifestyle behaviours, physical activity was no longer significantly associated with CVD risk markers. However, alcohol intake remained significantly and inversely associated with BMI (β = −0.412, *p* = 0.036) and waist circumference (β = −1.389 cm, *p* = 0.023).

### 3.5. Healthy Lifestyle Score

All participants had a HLS of at least one, as all were non-smokers. The percentage of participants scoring 1, 2, 3, 4 and 5 was 10.2%, 49.0%, 24.5%, 14.3% and 2.0% respectively. Table 1 presents participants socio-demographic characteristics, lifestyle behaviours and CVD risk markers, by HLS (1–5). There were no significant differences in socio-demographic characteristics by HLS. Linear regression analyses exploring the association between HLS and each CVD risk marker found no significant associations (Table 3).

## 4. Discussion

In this cross-sectional analysis, most young (18–35 years) overweight and obese women reported multiple unhealthy diet, alcohol, physical activity and sitting time behaviours. The majority of participants had a Healthy Lifestyle Score of two or less, with only one participant with the maximum score of five. Many of the participants also had adverse CVD risk markers, particularly waist circumference and plasma total, HDL and LDL cholesterol. The key association identified between lifestyle behaviour and CVD risks was that insufficient physical activity was associated with increased body fat, waist circumference and diastolic blood pressure. These findings suggest that targeting physical activity should be a priority for health interventions for this population. Overall the study participants were similar compared to the broader population of young adult women in Australia with respect to percentage meeting physical activity guidelines [9]; however, more study participants exceeded alcohol guidelines [44] and fewer were smokers [45]. Diet quality was similar to participants of a comparable Australian study in overweight and obese young adults [46]. More of the current study participants had elevated CVD risk markers, including higher percentages with elevated waist circumference [47] and total cholesterol [42], and low HDL cholesterol [42] than young Australian adults.

The current study found a significantly lower %body fat of 0.011% (*p* = 0.005), smaller waist circumference of 0.013 cm (*p* = 0.029) and lower diastolic blood pressure of 0.010 mmHg (*p* = 0.031) for every additional minute per week of moderate or vigorous physical activity. Despite these results only being significant in the unadjusted models, this association is consistent with previous cross-sectional and longitudinal studies demonstrating significant associations with higher physical activity and lower waist circumference and %body fat [48,49,50,51,52], including studies in young adult women [49,52]. Healy et al. also found higher physical activity to be associated with lower total: HDL cholesterol ratio and triglycerides, and increased HDL cholesterol [50]. These studies in middle-aged adults [48,50,51] also indicated a trend of lower blood pressure with increasing physical activity; however, statistical significance of the associations for systolic and diastolic blood pressure varied between the findings. The current study supports these findings, and additionally suggests that physical activity levels are impacting CVD risk markers in populations of younger overweight and obese women. Therefore, the findings provide further support for the need for physical activity interventions for young overweight and obese women.

Overall, the current study found no associations between diet quality or sitting time and CVD risk markers, and unexpectedly found an association between a higher alcohol intake and lower BMI, waist circumference and diastolic blood pressure. Conversely previous longitudinal studies of young and middle-aged women have reported associations between higher alcohol intake and increased hypertension and hypercholesterolaemia risk [22,53], as well as higher diet quality and lower hypertension risk [22] and weight gain [54]. Additionally previous cross-sectional studies have reported associations between higher sitting time and increased risk of higher waist circumference in young adult women [49] and overweight or obesity in young and middle-aged men and women [55,56]. The cross-sectional nature and inclusion criteria of the current study could partly explain the difference in findings. For example, the exclusion of healthy weight participants could have limited the comparative ability of the analyses. It is also possible that participants with higher BMI were trying to reduce alcohol intake, particularly as a means to achieve weight loss. Despite the current study findings regarding alcohol intake, the participants’ alcohol consumption is high and exceeds that of the wider young adult female population. As such health messages to reduce alcohol intake would be a pertinent addition to lifestyle interventions in this group.

The association between a higher number of high risk lifestyle behaviours and greater CVD risk has been demonstrated in several observational studies [17,18,19,20,21,22,25], including the cross-sectional analysis of young Australian adults by Gall et al. [25]. The current study found no associations between Healthy Lifestyle Score and CVD risk markers. With the exception of Gall et al. the other studies explored lifestyle behaviours in young adulthood and their combined impact on CVD risk markers 10 to 22 years later, hence the cross-sectional nature of the current analysis could account for the lack of significant findings. The HLS is a non-validated score, which was developed for the purposes of this analysis. There is a need for validated composite measures in this field that consider the relationship with health outcomes in large population samples. Other limitations of the study may also have impacted the results. The small sample size and the RCT inclusion/exclusion criteria may have limited the range of lifestyle behaviours and CVD risk markers assessed. Also, participants were enrolling in a weight loss study; they were not a random sample from the general population, which limits generalisability. The strict inclusion criteria may also have limited recruitment and the sample size as, despite formative research with the target group, recruitment and retention were lower than anticipated [26,57]. Additionally as a consequence of sample size, the study was not powered for all analyses. Other limitations should also be acknowledged when interpreting the results. As there are no current population-based recommendations for appropriate ARFS or sitting time the criteria for these were based on the distribution of the study participants’ data. Smokers were not represented, which may partially reflect the over representation of young women who were engaged in tertiary education studies [58]. Participants’ ability to recall dietary and alcohol intakes over a six-month period is potentially limiting. However, previous studies have used this time frame or longer [25], and the Australian Eating Survey has been validated over this time frame [27]. Finally, no data were obtained regarding participants’ family history of CVD or use of medications to treat elevated CVD risk factors. This information could have identified participants already at an increased risk of CVD. Future studies should obtain this information and include it in analyses as a confounder.

The strengths of the current study include the use of objective measures for all CVD risk markers (BMI, %body fat, waist circumference, blood pressure, plasma total, HDL and LDL cholesterol and triglycerides). Although some research has questioned the validity of bioelectrical impedance as a measure of body fat [59], the instrument used in the current study has comparative validity to dual-energy X-ray absorptiometry measures, and tends to underestimate rather than overestimate %body fat [60]. Secondly, the use of the AES FFQ, ARFS, Domain-Specific Sitting Time Questionnaire and Godin Leisure Time Exercise Questionnaire, all validated in middle and/or older-aged Australian women [27,28,32,61], reduces the potential bias from participants’ self-reporting these behaviours. In addition, the current study sample was representative of young adult women in Australia in terms of Aboriginality [62] and income [63].

## 5. Conclusions

The current study reinforces that unhealthy lifestyle behaviours and cardiovascular disease (CVD) risk markers are prevalent in young overweight and obese women. The major finding is that physical activity levels are directly associated with increased CVD risk markers. In addition, these young women are consuming high amounts of alcohol. This suggests that lifestyle interventions, especially those including physical activity, targeting young overweight and obese women are warranted and have the potential to improve cardiovascular risk factors and heart health across the adult life course.

## Figures and Tables

**Table 1:** Baseline characteristics of young (18–35 years) overweight and obese women (BMI 25–34.9 kg/m^2^) entering a weight-loss trial (*n* = 49 ^1^), by Healthy Lifestyle Score.

Variable	Healthy Lifestyle Score												*p*
	All participants		1		2		3		4		5		
	Mean or n (%)	SD	Mean or n (%)	SD	Mean or n (%)	SD	Mean or n (%)	SD	Mean or n (%)	SD	Mean or n (%)	SD	
N	49 (100.0)	-	5 (10.2)	-	24 (49.0)	-	12 (24.5)	-	7 (14.3)	-	1 (2.0)	-	-
Age (years)	27.1	4.5	27	5.2	27.4	4.3	27.8	4.7	25.5	5.2	22.1	-	0.56
Completed year 12 or equivalent	46 (93.9)	-	5 (100.0)	-	23 (95.8)	-	10 (83.3)	-	7 (100.0)	-	1 (100.0)	-	-
Currently studying	27 (55.1)	-	3 (60.0)	-	13 (54.2)	-	8 (66.7)	-	2 (28.6)	-	1 (100.0)	-	0.5
**Highest qualification**													0.35
Trade/Apprenticeship	2 (4.1)	-	0 (0.0)	-	1 (4.2)	-	1 (8.3)	-	0 (0.0)	-	0 (0.0)	-	-
Certificate/Diploma	13 (26.5)	-	0 (0.0)	-	9 (37.5)	-	3 (25.0)	-	1 (14.3)	-	0 (0.0)	-	-
University/Higher University Degree	21 (42.9)	-	4 (80.0)	-	8 (33.3)	-	4 (33.3)	-	5 (71.4)	-	0 (0.0)	-	-
**Ethnicity**													0.96
Australian	46 (93.9)	-	4 (80.0)	-	23 (95.8)	-	12 (100.0)	-	6 (85.7)	-	1 (100.0)	-	-
Other	3 (6.1)	-	1 (20.0)	-	1 (4.2)	-	0 (0.0)	-	1 (14.3)	-	0 (0.0)	-	-
Aboriginal origin	1 (2.0)	-	0 (0.0)	-	0 (0.0)	-	1 (8.3)	-	0 (0.0)	-	0 (0.0)	-	1.00
**Weekly income**													0.23
Lower ($0–$299)	12 (24.5)	-	1 (20.0)	-	2 (8.3)	-	6 (50.0)	-	2 (28.6)	-	1 (100.0)	-	-
Middle ($300–$999)	21 (42.9)	-	3 (60.0)	-	12 (50.0)	-	3 (25.0)	-	3 (42.9)	-	0 (0.0)	-	-
Higher (≥$1000)	16 (32.6)	-	1 (20.0)	-	10 (41.7)	-	3 (25.0)	-	2 (28.6)	-	1 (0.0)	-	-
**SES (IRSAD)**													0.64
1–3	11 (22.5)	-	1 (20.0)	-	6 (25.0)	-	3 (25.0)	-	1 (14.3)	-	0 (0.0)	-	-
4–6	22 (44.9)	-	3 (60.0)	-	10 (41.7)	-	6 (50.0)	-	2 (28.6)	-	1 (100.0)	-	-
7–9	16 (32.6)	-	1 (20.0)	-	8 (33.5)	-	3 (25.0)	-	4 (57.2)	-	0 (0.0)	-	-
ARFS	33.5	9.3	24.6	5.3	31.8	9.1	35.9	8.7	40.6	7.2	42.0	-	0.01
Top quintile (≥40)	12 (24.5)	-	0 (0.0)	-	2 (8.3)	-	4 (33.3)	-	5 (71.4)	-	1 (100.0)	-	-
Alcohol intake (standard drinks/day)	3.3	2.4	4.0	1.0	3.3	2.6	3.1	2.2	3.1	3.1	2.0	-	0.63
Meeting HLC	27 (55.1)	-	0 (0.0)	-	14 (58.3)	-	7 (58.3)	-	5 (71.4)	-	1 (100.0)	-	-
Total physical activity (min/week)	207	225	42	61	145	155	282	188	399	391	300	-	0.003
Meeting HLC	25 (51.0)	-	0 (0.0)	-	8 (33.3)	-	9 (75.0)	-	7 (100.0)	-	1 (100.0)	-	-
**Sitting time (min/day)**													-
Weekday	610	260	530	158	722	274	538	234	433	175	450	-	0.03
Weekend day	499	200	548	119	548	240	438	131	416	174	360	-	0.42
Average of days	578	213	535	95	672	230	509	174	428	152	424	-	0.01
Lowest quintile (≤394)	9 (18.4)	-	0 (0.0)	-	0 (0.0)	-	4 (33.3)	-	4 (57.1)	-	1 (100.0)	-	0.06
Non-smokers	49 (100.0)	-	5 (100.0)	-	24 (100.0)	-	12 (100.0)	-	7 (100.0)	-	1 (100.0)	-	-
BMI (kg/m^2^)	29.4	2.6	29.9	2.9	29.2	2.7	28.4	2.0	31.8	1.5	28.9	-	0.08
Overweight	27 (55.1)	-	2 (40.0)	-	14 (58.3)	-	9 (75.0)	-	1 (14.3)	-	1 (100.0)	-	-
Obese	22 (44.9)	-	3 (60.0)	-	10 (41.7)	-	3 (25.0)	-	6 (85.7)	-	0 (0.0)	-	-
Body fat %	37.7	5.7	39.4	7.6	37.5	5	35.7	6.5	40.2	4.9	40.3	-	0.57
Waist circumference (cm)	88.8	8.6	88.2	6.9	88.4	9.3	87.5	8.4	91.5	9.1	96.8	-	0.60
≥80 cm	17 (34.7)	-	3 (60.0)	-	10 (41.7)	-	3 (25.0)	-	1 (14.3)	-	0 (0.0)	-	-
≥88 cm	24 (49.0)	-	2 (40.0)	-	10 (41.7)	-	6 (50.0)	-	5 (71.4)	-	1 (100.0)	-	-
Systolic blood pressure (mmHg)	112.1	8.6	116.4	7.4	112.9	8.5	109.4	8.9	112	9.9	107	-	0.35
Diastolic blood pressure (mmHg)	74.3	7.8	76.6	4.5	75.1	9.5	71.3	5.1	75.7	6.4	69	-	0.26
Total cholesterol (mmol/L)	5.0	0.9	4.7	0.6	5.1	0.9	5.2	1.0	5.1	1.2	4.2	-	0.73
≥5.5 mmol/L	16 (33.3)	-	1 (20.0)	-	7 (29.2)	-	5 (45.5)	-	3 (42.9)	-	0 (0.0)	-	-
HDL-C (mmol/L)	1.5	0.4	1.3	0.3	1.6	0.5	1.5	0.5	1.4	0.4	1.4	-	0.63
<1.3 mmol/L	17 (38.6)	-	4 (80.0)	-	7 (33.3)	-	3 (30.0)	-	3 (42.9)	-	0 (0.0)	-	-
LDL-C (mmol/L)	2.9	0.8	2.9	0.7	2.8	0.7	3	0.7	3.1	1.0	2.3	-	0.89
≥3.5 mmol/L	10 (22.7)	-	1 (20.0)	-	4 (19.1)	-	2 (20.0)	-	3 (42.9)	-	0 (0.0)	-	-
Triglycerides (mmol/L)	1.2	0.5	1.1	0.1	1.2	0.6	1.3	0.4	1.2	0.4	1.2	-	0.93
≥2.0 mmol/L	2 (4.2)	-	0 (0.0)	-	1 (4.2)	-	1 (9.1)	-	0 (0.0)	-	0 (0.0)	-	-

BMI, body mass index; SES, socio-economic status; IRSAD, Index of Relative Socio-Economic Advantage and Disadvantage; ARFS, Australian Recommended Food Score; HLC, Healthy Lifestyle Criteria; HDL-C, high density lipoprotein cholesterol; LDL-C, low density lipoprotein cholesterol. ^1^ HDL and LDL analysis conducted on 44 participants; TC and TG analysis conducted on 48 participants.

**Table 2:** Linear regression analysis results of lifestyle behaviours with CVD risk markers in young (18–35 years) overweight and obese women (BMI 25–34.9 kg/m^2^) entering a weight-loss trial.

Variable	Unadjusted Model ^1^			Full-Adjusted Model ^2^		
	β-Coefficient ^3^	SE	*p*	β-Coefficient	SE	*p*
BMI (kg/m^2^)						
ARFS	0.014	0.051	0.780	0.087	0.054	0.113
Alcohol intake (standard drinks/day)	−0.458	0.174	**0.012** *	−0.412	0.188	0.036^*^
Physical activity (min/week)	−0.003	0.002	0.073	−0.004	0.002	0.124
Average sitting time (min/day)	0.001	0.002	0.664	−0.001	0.002	0.766
Body fat (%)						
ARFS	−0.108	0.103	0.303	0.035	0.111	0.757
Alcohol intake (standard drinks/day)	−0.735	0.373	0.056	−0.410	0.391	0.301
Physical activity (min/week)	−0.011	0.004	**0.005** *	−0.009	0.005	0.065
Average sitting time (min/day)	0.006	0.004	0.143	0.003	0.004	0.523
Waist circumference (cm)						
ARFS	−0.170	0.157	0.286	0.037	0.167	0.825
Alcohol intake (standard drinks/day)	−1.673	0.529	**0.003** *	−1.389	0.586	0.023^*^
Physical activity (min/week)	−0.013	0.006	**0.029** *	−0.009	0.007	0.210
Average sitting time (min/day)	0.002	0.007	0.800	−0.003	0.006	0.671
Systolic blood pressure (mmHg)						
ARFS	−0.001	0.001	0.375	−0.002	0.185	0.989
Alcohol intake (standard drinks/day)	−0.004	0.005	0.459	−0.187	0.650	0.775
Physical activity (min/week)	−0.0001	0.0001	0.274	−0.010	0.008	0.193
Average sitting time (min/day)	−0.0001	0.0001	0.165	−0.013	0.007	0.074
Diastolic blood pressure (mmHg)						
ARFS	−0.210	0.126	0.103	−0.064	0.141	0.653
Alcohol intake (standard drinks/day)	−0.988	0.463	**0.039** *	−0.653	0.496	0.197
Physical activity (min/week)	−0.010	0.005	**0.031** *	−0.008	0.006	0.165
Average sitting time (min/day)	−0.002	0.005	0.758	−0.005	0.005	0.342
Total cholesterol (mmol/L) ^4^						
ARFS	−0.017	0.016	0.288	0.001	0.001	0.267
Alcohol intake (standard drinks/day)	0.046	0.058	0.432	−0.002	0.003	0.389
Physical activity (min/week)	−0.00004	0.001	0.941	0.00001	0.00003	0.788
Average sitting time (min/day)	−0.0003	0.001	0.626	0.00002	0.00003	0.545
HDL cholesterol (mmol/L) ^4^						
ARFS	0.002	0.008	0.851	-0.007	0.010	0.483
Alcohol intake (standard drinks/day)	0.045	0.034	0.199	0.038	0.039	0.334
Physical activity (min/week)	0.0004	0.0003	0.250	0.0005	0.0004	0.275
Average sitting time (min/day)	0.0001	0.0004	0.724	0.0004	0.0004	0.364
LDL cholesterol (mmol/L) ^4^						
ARFS	−0.006	0.005	0.175	−0.015	0.016	0.338
Alcohol intake (standard drinks/day)	−0.006	0.020	0.781	−0.009	0.062	0.889
Physical activity (min/week)	−0.0001	0.0002	0.714	−0.00005	0.001	0.934
Average sitting time (min/day)	−0.0003	0.0002	0.199	−0.001	0.001	0.194
Triglycerides (mmol/L) ^4^						
ARFS	0.007	0.005	0.169	0.004	0.005	0.516
Alcohol intake (standard drinks/day)	0.005	0.018	0.761	−0.009	0.019	0.648
Physical activity (min/week)	0.0003	0.0002	0.086	0.0003	0.0002	0.217
Average sitting time (min/day)	−0.0001	0.0002	0.737	0.00004	0.0002	0.865

CVD, cardiovascular disease; BMI, body mass index; SE, standard error; ARFS; Australian Recommended Food Score; HDL cholesterol, high density lipoprotein cholesterol; LDL cholesterol, low density lipoprotein cholesterol. ^1^ The unadjusted model includes the individual lifestyle behaviour and age, ethnicity and socio-economic status; ^2^ The fully adjusted model includes all lifestyle behaviours (diet quality, alcohol intake, physical activity, sitting time) and age, ethnicity and socio-economic status; ^3^ β-Coefficient indicates the increase in the CVD risk marker per unit increase in the independent variable; ^4^ HDL and LDL cholesterol analysis conducted on 44 participants; Total cholesterol and triglycerides analysis conducted on 48 participants; * Indicates statistically significant result (*p* < 0.05).

**Table 3:** Linear regression analysis results for CVD risk markers with Healthy Lifestyle Score in young (18–35 years) overweight and obese women (BMI 25–34.9 kg/m^2^) entering a weight loss trial (*n* = 49 ^1^).

Variable	β-Coefficient ^2^	SE	*p*
BMI (kg/m^2^)	0.419	0.464	0.372
Body fat (%)	−0.542	0.965	0.577
Waist circumference (cm)	0.695	1.468	0.639
Systolic blood pressure (mmHg)	0.0001	0.0001	0.283
Diastolic blood pressure (mmHg)	−1.236	1.378	0.375
Total cholesterol (mmol/L)	0.027	0.147	0.856
HDL cholesterol (mmol/L)	−0.047	0.079	0.550
LDL cholesterol (mmol/L)	0.009	0.045	0.851
Triglycerides (mmol/L)	0.019	0.053	0.729

CVD, cardiovascular disease; BMI, body mass index; SE, standard error; HDL cholesterol, high density lipoprotein cholesterol; LDL cholesterol, low density lipoprotein cholesterol. ^1^ HDL and LDL cholesterol analysis conducted on 44 participants; total cholesterol and triglycerides analysis conducted on 48 participants; ^2^ β-coefficient indicates the increase in the CVD risk marker per unit increase in the independent variable; ^3^ Models adjusted for age, ethnicity and socio-economic status.
